# The reliability and validity of a portfolio designed as a programmatic assessment of performance in an integrated clinical placement

**DOI:** 10.1186/1472-6920-14-197

**Published:** 2014-09-20

**Authors:** Chris Roberts, Narelle Shadbolt, Tyler Clark, Phillip Simpson

**Affiliations:** Sydney Medical School - Northern, University of Sydney, Hornsby Ku-ring-gai Hospital, Palmerston Road, Sydney, NSW 2077 Australia; Office of Medical Education, Sydney Medical School, University of Sydney, Sydney, NSW 2006 Australia

**Keywords:** Portfolio, Programmatic assessment, Competency-based assessment, Clinical placement, Longitudinal integrated clerkship, Generalisability theory, Reliability, Validity

## Abstract

**Background:**

Little is known about the technical adequacy of portfolios in reporting multiple complex academic and performance-based assessments. We explored, first, the influencing factors on the precision of scoring within a programmatic assessment of student learning outcomes within an integrated clinical placement. Second, the degree to which validity evidence supported interpretation of student scores.

**Methods:**

Within generalisability theory, we estimated the contribution that each wanted factor (i.e. student capability) and unwanted factors (e.g. the impact of assessors) made to the variation in portfolio task scores. Relative and absolute standard errors of measurement provided a confidence interval around a pre-determined pass/fail standard for all six tasks. Validity evidence was sought through demonstrating the internal consistency of the portfolio and exploring the relationship of student scores with clinical experience.

**Results:**

The mean portfolio mark for 257 students, across 372 raters, based on six tasks, was 75.56 (SD, 6.68). For a single student on one assessment task, 11% of the variance in scores was due to true differences in student capability. The most significant interaction was context specificity (49%), the tendency for one student to engage with one task and not engage with another task. Rater subjectivity was 29%. An absolute standard error of measurement of 4.74%, gave a 95% CI of +/- 9.30%, and a 68% CI of +/- 4.74% around a pass/fail score of 57%. Construct validity was supported by demonstration of an assessment framework, the internal consistency of the portfolio tasks, and higher scores for students who did the clinical placement later in the academic year.

**Conclusion:**

A portfolio designed as a programmatic assessment of an integrated clinical placement has sufficient evidence of validity to support a specific interpretation of student scores around passing a clinical placement. It has modest precision in assessing students’ achievement of a competency standard. There were identifiable areas for reducing measurement error and providing more certainty around decision-making. Reducing the measurement error would require engaging with the student body on the value of the tasks, more focussed academic and clinical supervisor training, and revisiting the rubric of the assessment in the light of feedback.

## Background

Representative collections of work assembled in a portfolio are widely used in all stages of health professional education from university through to vocational and professional programmes. A number of reviews have illustrated the varying definitions and differences in their use [1, 2]. There are broadly two purposes for portfolio assessment in medical and health science education. The first is for formative assessment [3], with a commitment to critical reflection as the dominant learning and teaching process, and content, which addresses many matters of personal and professional development. The purpose of the assessment is for enhancing student learning, and in order to do so, it is claimed portfolios must include explicit and mandatory critical self-reflection [4, 5] and the emphasis of assessment is feedback on student self-reflection through qualitative means [6, 7].

In contrast, an alternative portfolio approach, and the one taken in this research, aims to provide a comprehensive, competency-based assessment that is fully integrated with the curriculum. This can include reflection as one of many desired competencies [8]. Well established in some North American and European settings, this type of portfolio both supports student learning and assesses a range of competencies including; basic science, clinical and population health knowledge, communication (both oral and written) skills, clinical and procedural skills, and appropriate professional behaviours such as self-reflection, empathy, ability to work within teams, and motivation for lifelong learning [9]. Research on the reflective portfolio tends to dominate the literature. Buckley *et al*. [1] in their survey of health professional education portfolios noted that, whilst most were of the reflective type, at least a quarter where of the competency type.

Given the lack of consensus on what the purpose of a portfolio assessment is, an alternative theoretical approach to assessing multiple samples of students’ work was developed by Maastricht University. Their integrated programmatic assessment approach [10] aims to provide a comprehensive assessment of student learning across a diverse range of intended outcomes, free from a commitment to any particular instructional approach. A rich picture of student learning is developed through multiple sources of assessment including qualitative data. However, instituting programmatic assessment is challenging within an environment largely favouring a psychometric approach to summative assessment [10]. We contend that first, competency-based portfolios are a form of programmatic assessment in that they aggregate complex assessments of student performance in a meaningful way for both staff and students. Second that a programmatic portfolio has a number of advantages in assessing the diverse learning outcomes typical of integrated clinical placements. There are challenges to using portfolios in this context. A major one lies in establishing a validated framework that provides some assurance that student scores would not be reversed or substantially altered, if a second independent assessment of the portfolio were made*.* Further, the assessment should have a technical adequacy to justify any decision that penalises a low scoring student [11], particularly where there are consequences attached to passing and failing decisions. It had been thought that a high level of reliability was critical where the main purpose of the portfolio was for high stakes decision-making [3]. However this statement is now problematic given the lack of consensus internationally as to what constitutes technical adequacy [11] in the psychometric analysis of aggregates of complex performance assessments. In medical education, this has been traditionally established through reliability and validity research.

### Reliability

Portfolio-based assessments need to demonstrate that students’ scores are within a margin of error small enough so as to make the effort and use of resources of the assessment worthwhile [11]. From the perspective of generalisability theory, signal comes from the student capability and noise comes from a number of variables and their interactions including the number of assessors, the number of portfolio tasks, as well as the process of combining the differing tasks. The ratio of signal to noise has been traditionally reported as reliability co-efficient.

#### Judges

An acceptable level of reliability for high stakes assessment has been traditionally set at 0.8 [12], although in clinical settings r = 0.7 is considered more feasible. The number of judges required to achieve the desired 0.8 reliability level, has varied across the literature. For example, a systematic review found an average inter-rater reliability of 0.63 [9]. If the number of assessors per portfolio increased to 2, 3, or 4 then the reliability would increase to 0.77, 0.84, and 0.87 respectively. There is also the known issue of high levels of variability in judging performance related tasks, which appears to be resistant to training. Ensuring individual judges marked portfolios, for example, by two tasks only, would limit the impact of assessors to particular tasks, and thus improve reliability [13]. Recent research has examined the issue of rater cognition [14–16] in order to understand, and perhaps train for, the underlying cognitive processes that raters use when judging the abilities of their learners. However even less is known about the performative schema that judges might use across a range of disparate tasks, and whether they can judge more than one type of task in a portfolio independently.

#### Tasks

Another variable affecting reliability is the number of tasks that the students were asked to include in their portfolios. One paper found that 13 tasks were necessary when one rater was used, or 9 tasks if two raters were used [17]. Another paper determined that 5 tasks were sufficient for relative decisions when two raters were used but that three raters were needed for absolute decisions at this task level [18]. In order to stay within acceptable student workload requirements, increasing the number of tasks for the sake of improving reliability may decrease the utility of the assessment. Validity will be decreased through trivialising tasks, and feasibility compromised because of resource implications of finding enough assessors. The most significant and commonly found interaction contributing to measurement noise is that between student and task, a form of context specificity [2]. However, there is relatively little published work providing explanatory frameworks as to how this might be best accounted for both technically [19] and interpretatively, particularly in aggregates of diverse assessments. A presiding assumption in the literature is that the student task interaction is about student ability, across tasks. However the broader educational literature suggests that much of the degree to which students achieve learning outcomes relates to their degree of motivation and engagement in the task [20]. Thus a large student task interaction may represent unreliability of the assessment program, variation in engagement, or ability across varying tasks, or diversity in the psychological constructs being assessed.

### Validity

Messick [21] defined validity as “an integrated evaluative judgment of the degree to which empirical evidence and theoretical rationales support the adequacy and appropriateness of inferences and actions based on test scores and other modes of assessment”. Validity is a judgment about the degree to which each component of the portfolio assessment system; which includes content coverage, response processes, internal structure, and relations to external variables, are clearly defined and adequately implemented. As regards what content portfolios are assessing, traditional assessments are set by teaching staff, which allows control over the material covered and standardisation in assessing student performance. Portfolios rely on student-driven submissions that may cover a range of topics set by teachers. Thus it is important to determine if portfolios are assessing the knowledge required of students to gain competency in relation to the expected learning outcomes. Post-portfolio evaluation at Dundee Medical School [22] found that examiners acknowledged the portfolio’s ability to reveal student strengths and weaknesses and demonstrated the examiners’ confidence in the portfolio process to accurately assess the students in the program. Overall, students felt that the portfolio heightened their learning and their comprehension of the institutional learning outcomes [23]. Driessen *et al*. concluded that portfolio tasks in medical school were effective in not only assessing students’ reflective skills but in developing them as well [7].

Roberts *et al*. noted some evidence for construct validity of postgraduate reflective portfolios [13] in that an assessment tool was able to distinguish between good, satisfactory and poor General Practitioner (GP) reflective portfolios, as well as having good internal consistency. There is also the timing of the portfolio assessment within the year of training. For example portfolio-based assessment in evaluating the competency of psychiatric residents found a general trend of increasing scores with the stage of training [17] which lends support to the portfolio as having construct validity [24], as learners perform better after more experience.

### Precision in achieving a standard

A significant challenge for portfolio assessment is that most psychometric rules apply to a single format [11]. There are few frameworks published for combining several formats to arrive at a composite score. A number of processes have been pragmatically applied by assessment committees, including simply adding task scores, converting raw scores to a normalised scale and then adding together, and employing a series of weightings. Conventional test analysis has centred on the errors in reporting individual differences in ability, reporting a “reliability coefficient” as the summary of a test’s accuracy, which is traditionally reported as a ratio of non-error variance to total variance. Cronbach *et al*. [11] were sceptical of the utility of such a measure in complex performance-based assessments, because “of the magic number of 0.80” that a reliability coefficient should supposedly reach.

Cronbach *et al*. noted that individual differences in student scores are beside the point in comparisons against a standard. [11] Contemporary assessments should describe the performance of each student in absolute terms, with a particular score reflecting the achievement of a standard. This requires a different examination of measurement error. The absolute standard error of measurement (SEM) is an appropriate indicator of uncertainty, which provides a confidence interval around a student’s score. It indicates how much fluctuation would be likely if repeated assessment could collect many scores for the same student or aggregate [11]. To calculate the SEM, when the aim is to locate the individual’s absolute level on the score scale, one adds all error contributions and takes the square root of the sum. For a relative SEM, the task component does not enter the calculation, because a comparative report of student marks is not affected by the difficulty of a task given to all students. Likewise, the rater component and the task rater interaction. This can result in a relative standard error of measurement some 15% lower than an absolute SEM depending on the purpose of the portfolio [11].

Alternatively, van der Vleuten and Schuwirth [10, 25] have suggested that providing rich qualitative insights from expert assessors to the students can enhance validity. It remains unclear how this could be achieved practically, or how defensible decisions could be made. However there is little doubt that for many schools, there is a lack of resources to provide detailed qualitative feedback to such rigorous quality criteria, whist at the same time conforming to the psychometric expectations of standard setting and decision making. The attraction remains of examining whether a portfolio conceived as a programmatic assessment can have reasonable psychometric properties, in particular precision around the pass/fail standard. Second, if assessment committees can understand where the major sources of error are coming from, they can begin to address these in order to refine the assessment for future iterations.

### Context

An opportunity to further investigate these issues arose in an eight-week community clinical placement (clerkship in North America) in the third and fourth years of a four-year graduate entry medical program. The assessment was of the programmatic variety, and student learning was immersive and community-based [26], features of a longitudinal integrated clinical placement (LIC). Learning and teaching in the community presents both opportunities and challenges for curriculum design and ensuring the validity of the assessment system in terms of content coverage and marking processes [21]. Although most health care encounters take place in the community and increasingly students are learning in this environment, medical education has traditionally taken place in more controlled environments [27]. In our community setting, students were widely dispersed geographically across urban, rural, remote, and international general practices, and thus self-directed learning was paramount. Since, the perceived quality of general practice placements is determined by the quality of supervision [28], there had been extensive engagement with the network of GP supervisors providing placements. The assessment had to take account of this context and reflect the knowledge, skills and professional behaviours required by the medical program.

Students received an academic orientation, an immersive experience in primary and community care (3 weeks in an urban practice and 4 weeks rural) with general practitioners acting as supervisors and assessors. They were briefed on self-directed learning, and a primary care approach to clinical reasoning [29]. The specific content was driven by national priorities in managing chronic care conditions in primary care (http://www.aihw.gov.au/national-health-priority-areas/), locally harvested data about the prevalence of specific and common problems [30], and learning outcomes aligned with the major primary care vocational training groups [31, 32]. The portfolio-based assessment was constructed to reflect the integrated nature of the placement across the contexts of urban family practice, rural medicine, indigenous community health, culturally diverse populations, and global health. It focused students’ attention in the areas of evidence based decision-making, social determinants of health, clinical reasoning, student-as-educators, clinical skills, professionalism, and extending their clinical knowledge base. Opportunities for critical self-reflection [5] were promoted. The assessment process was supported by the medical school on-line learning management system, to provide orientation materials, student guides, supervisor guides, and a marking system for academic supervisors. The portfolio included a range of assessments, self-directed learning tasks, and required learning activities, which together formed a programmatic portfolio assessment. Six of these assignments were awarded marks, which were used to derive a final portfolio mark. As per the medical school assessment strategy, all students deemed to have scored less than the pass/fail mark undertook a supplementary assessment to determine the right to progress to the next specialty placement.

The aim of this research was to explore

-  The influencing factors on the precision of scoring within a portfolio-based assessment of students’ achievement of primary and community care learning outcomes within an eight week integrated clinical placement.
-  The degree to which evidence of validity (content coverage, the marking processes, and the internal structure), were clearly defined and supported the interpretation of the student scores.

## Methods

### A programmatic assessment portfolio

The six tasks in the portfolio that were summatively assessed were: an Evidenced Based Medicine (EBM) Task, a Community Profile Project (CCP), a Primary Care Areas of Priority Case (PCAP) [33], two GP supervisor assessments (one Urban, one Rural), and a Written Summative Assessment consisting of Multiple Choice Questions (MCQ). The details and their weighted contribution to the overall portfolio mark are given in Table 1. Acknowledging that reflection when learning from a clinical experience is a core skill for professional development [9], additional tasks were included in the clinical placement but were not marked summatively. These included a small group reflection on a critical incident from practice, and opportunities for student self-assessment prior to both GP supervisors’ reports.

**Table 1:** **Individual tasks making up the community placement programmatic portfolio assessment**

1.	*Evidenced Based Medicine (EBM) Task -* (20% of the portfolio mark) The EBM marking criteria had 15 assessable components assigned a score of 1-4 by the marker, with a maximum total score of 60. In a written assignment, students identified a health related question from a patient they had seen in general practice, and then applied an evidence based practice approach in order to provide a patient narrative that answered the patient’s question.
2.	*Community Profile Project* (*CCP) -* (20%) The CCP task had 9 assessable components, mostly scored 1-4 (two items were marked out of 2) for a maximum total of 34. A further written assignment where the student needed to explore and identify important sub-groups within a chosen community, describe and understand what the major health issues were, and consider what some of the determinants of health, and additional services might be for such a community.
3.	*Primary Care Areas of Priority Cases (PCAPs) -* (10%) The PCAP task had 11 assessable components, scored 1-4, for a maximum total of 44. Each student in the cohort presented an interactive one-hour case-based teaching session to a small group of their peers derived from one of eight national health priority areas.
4. and 5.	*Two Supervisor Assessments (both an Urban and Rural Assessment)* – (10%) each. The Supervisor tasks had 9 assessable components, eight of which were scored 1-4, and one (punctuality) on a scale of 1-3 giving a maximum total of 35 each. The supervisor assessed the students on communication skills history taking, examination skills, clinical reasoning, investigation and management plans, preventative health, professional behaviours.
6.	*Written Summative Assessment (MCQ)* – (30%) This was a 60-item single best answer written assessment in which the material for the assessment came from the PCAPS. (largely chronic disease management problems) and pre-prepared self directed learning problems (largely acute common primary care problems).

### Marking rubric

Each of the assessment tasks, excluding the MCQ, was marked on a four-point scale, to encourage standardisation across raters about the expected level of student performance. The criteria checklist items were specifically related to the particular task. Scores were converted to raw percentages and then combined in accordance to the pre-determined weightings to give a final portfolio mark. A modified Angoff was undertaken with at least five academic assessors to determine the pass/fail cut score for each individual task and for the aggregated total portfolio score.

### Distribution of students

To fit in with timetabling of all the specialty clinical placements, the Community placement was undertaken in four streams labelled B-E, in any one academic year.

### Student and staff orientation

In the first week of the placement, students were prepared for the nature of the assessment, and orientated towards the model of clinical reasoning in general practice. The requirements of each assessment task were provided in overview. Staff training consisted of written materials, which were made available on the medical school website, a video conferenced workshop, and a clinical school site workshop lead by the second author.

### Ethics

An existing collection of assessment data that was de-identified was used for this analysis, which fulfilled the Sydney University Human Research Ethics Committee (HREC) criteria of negligible risk, and therefore did not require formal ethical approval.

### Statistical analysis

In Generalisability theory [34], the G–study provided a means of quantifying the sources of potential error in the assessment simultaneously, using all of the data available. The student’s universe score consists of all the trials of the assessment design that might hypothetically be carried out, using innumerable sets of tasks, administered on distinct occasions, with innumerable scorings of each performance by qualified scorers [11].

A variance components analysis estimated the contribution that each wanted factor (i.e. the capability of the student) and unwanted factors (e.g. the impact of the assessor) made to the variation in portfolio task scores. Variance estimates were then combined [35] to provide an index of reliability (the G coefficient), and relative and absolute standard errors of measurement [11]. The strength of this approach was that future modification of the assessment program could be planned that address the main sources of error identified in the initial study. We used the General Linear Model within SPSS (version 20) to undertake the G-Study using a partially crossed model [35]. Students were fully crossed with tasks. Assessors were partially crossed with tasks in that academic supervisors (n = 15-32) marked from one to three written tasks from the EBM, CCP, and PCAPs four times in a year. Similarly GP supervisors (n = 85) had marked up to four students in the year on either an urban or on a rural GP clinical placement. Some GP supervisors also marked academic assessments. The MCQ was machine marked. Given that there was sufficient crossing in the data to run the General Linear Model, we chose to use the partially crossed design to maximise the information from variance estimates. The subsequent D study modelled changes in reliability and the standard error of measurement commensurate with increasing the numbers of tasks. Cronbach’s alpha and Pearson’s coefficient for inter-item correlations were used to determine the assessment program’s internal consistency, as part of the validity evidence.

## Results

Portfolio assessment data was available for 257 of the 260 students (98.8%) who completed the Community Integrated Clinical Placement in an academic year. Of the 3 students who were not included, two had incomplete assessment data due to deferral and/or withdrawal, while one student was repeating the placement for revision purposes. Overall 15 to 32 different academics marked the EBM, Community Profile or PCAPs in each of the four streams making up the placement. In total, 340 (178 urban and 162 rural) differing General Practitioners assessed the students during the urban and rural placements.

Aggregated marks for the 257 portfolios were normally distributed with a mean of 75.56 and a standard deviation of 6.68, with a minimum mark of 50.6 and maximum of 90.2. The pass/fail cut score for the portfolio was set at 57% following the modified Angoff procedure.

Table 2 presents descriptive statistics for the raw percentages of the 6 tasks, and the weighted total score of the integrated portfolio assessment (excluding missing assignments; n = 2 for both EBM and CCP). Both Supervisor Assessments had the highest mean raw per cent of the assessments, while the Written Summative (MCQ) had the lowest (66.51%). All assessment distributions showed a negative skew, as the assessments were criterion-based and assessed a relatively homogenous cohort of medical students.

**Table 2:** **Descriptive statistics for portfolio assessment of a community placement**

Portfolio task	N	Minimum	Maximum	Mean	Std. deviation
EBM	255	35	100	77.15	11.56
PCAP	257	48	100	79.50	10.70
CCP	255	40	100	81.16	10.55
Supervisor-urban	257	37	100	83.28	11.51
Supervisor-rural	257	43	100	85.53	11.79
MCQ	257	39	88	66.51	8.66
Overall portfolio mark	257	50.6	90.2	75.56	6.68

### Reliability

Together, the unweighted percentage scores for each of the six tasks, which comprised the programmatic assessment, were moderately internally consistent, with a Cronbach’s α (r = 0.52). The corrected item-total correlation, the degree to which each component of the integrated assessment contributed to the total score, showed that all components were positively correlated, albeit modestly (r = 0.17 - 0.39), with the population health orientated CCP having the lowest correlation of 0.17.

The analysis of variance components in the G-study (see Table 3) showed that for one assessor and one portfolio task, the variance due to factors related to the student was 11%. The single largest source of construct irrelevant error (49%), was due to context specificity, the interaction between student and the task, reflecting the varying performance of students between portfolio tasks (across assessors). The second largest error source was rater subjectivity 29%, with 14% reflecting the stringency or leniency of the assessor, 11% reflecting the stringency/leniency for a specific task over and above their usual stringency/leniency and 4% the varying views that assessors have of the student’s capability. Unwanted variance due to the difficulty or ease of tasks was 12%.

**Table 3:** **Variance components from partially crossed generalisability study**

Component and their interaction	Explanation of interactions	Variance components	%
**Student**	The variance in marks due to true differences in the capability of the student	17.18	11.28
**Task**	The consistent tendency for one task to be marked higher or lower than others	17.81	11.70
**Assessor**	The tendency for one assessor to score a task highly and another to score the same task poorly	21.13	13.88
**Assessor with student**	The varying views that assessors have of students capability	5.60	3.68
**Assessor with task**	The tendency for an assessor to mark a task higher or lower in addition to their usual stringency/leniency	16.10	10.58
**Student with task**	The tendency for the student to engage with one task and not another	74.42	48.88
**Residual error**	Residual variation not explained by other factors	0.00	0.00

Using these variance components, the D-study estimated the reliabilities of the assessment with varying numbers of tasks, and increasing the number of raters per task. In Table 4, by increasing the number of tasks, the reliability of the judgement in relation to the portfolio increases. If we accept the lower level of 0.7 on the grounds of feasibility, then 20 tasks would be needed, a figure significantly greater than found previously [17].

**Table 4:** **Decision study modelling changes in reliability co-efficient, relative and absolute standard of errors of measurement when increasing the numbers of tasks**

Tasks	Reliability coefficient	Relative SEM	Relative 95% CI	Absolute SEM	Absolute 95% CI
20	0.72	2.00	3.92	2.60	5.09
16	0.67	2.24	4.38	2.91	5.69
12	0.60	2.58	5.06	3.35	6.58
10	0.56	2.83	5.54	3.68	7.20
8	0.50	3.16	6.20	4.11	8.05
6	0.43	3.65	7.16	4.74	9.30
4	0.34	4.47	8.77	5.81	11.39

### Decision making around achieving the standard

Absolute standard errors of measurement were calculated (See Table 4) in order to locate the student’s absolute level on the score scale. For illustrative purposes relative SEMs were calculated with the task component removed, because a comparative report is not affected by the difficulty of a task given to all students [11]. For a portfolio of six tasks, the absolute SEM is 4.74%. For a pass/fail standard score of 57%, this SE would give a 95% CI (9.30%) that the student’s true score would lie between 47.70% to 66.30%. Thus students falling below 47.70% would only have a one in twenty chance of being misclassified as a fail. Applying a 68% CI (4.74%) would mean that students falling below 52.26% would have a one in three chance of being misclassified as a fail.

### Validity

Building on the integrated approach to validity of Messick [21] and others, Downing asserts that assessments are not valid or invalid; rather, the scores or outcomes of assessments have more or less evidence to support (or refute) a specific interpretation of student scores (such as passing or failing a course) [36]. Most tasks within the portfolio showed a low-moderate significant positive correlation with one another (see Table 5). The EBM Task was significantly correlated with all assessments and most so with the knowledge-based MCQ (r = 0.34). The PCAP was not significantly correlated with the MCQ, nor was the rural supervisor assessment (r = 0.03) despite the urban supervisor assessment being significantly correlated (r = 0.21). Not surprisingly, both supervisor assessments were correlated with one another (r = 0.33).

**Table 5:** **Pearson’s correlations of community placement portfolio tasks (N = 257)**

	PCAP	CCP	Supervisor urban	Supervisor rural	MCQ
EBM	0.33^**^	0.16^**^	0.19^**^	0.11	0.17^**^
PCAP	1	0.16^*^	0.14^*^	0.12^*^	0.11
CCP		1	0.02	- 0.02	0.23^**^
Supervisor-urban			1	0.33^**^	0.21^**^
Supervisor-rural				1	0.03

**Significant at p < 0.001.

*Significant at p < 0.05.

Stream B, the first time Year 3 students undertake a specialty clinical placement, was shown to have the lowest overall mean of 70.37% (see Table 6, while Stream E had the highest (mean = 78.44%). An analysis of variance (ANOVA) was performed to detect possible differences in assessment performance between streams. Levene’s test of homogeneity of variance was not significant (p = 0.72) indicating that the variance was approximately equal between groups. The ANOVA indicated a significant difference between streams in overall assessment performance (F = 24.07, p < 0.001). A Tukey post-hoc analysis identified a significant difference between Stream E and all other streams (p = <0.001), with Stream B students having a significantly lower overall portfolio mark by approximately 7%.

**Table 6:** **Community placement portfolio scores, means, and standard deviations in order of Stream**

Stream	N	Mean	Standard deviation	Minimum	Maximum
B	67	70.37	5.73	50.6	80.8
C	71	76.69	6.21	53.9	90.2
D	69	77.34	5.96	60.1	90.1
E	50	78.44	5.72	63.5	89.5

Those students in Stream B could be disadvantaged due to a lack of clinical experience, compared with Stream E students, who bring a greater wealth of background knowledge and experience to the placement. This concurs with the findings of others in postgraduate settings [10, 17, 24] and provides further evidence of construct validity.

## Discussion

Our findings illustrate the methodological issues of reporting multiple assessments from an integrated clinical placement in a programmatic portfolio. It demonstrates that this type of portfolio does have sufficient technical adequacy to justify decisions that might penalise a low scoring student for not having achieved the required standard. In particular, these findings demonstrate that within the eight-week community clinical placement explored in this research, a level of precision around the pass/fail standard could be determined. It was possible to put an absolute 95% confidence interval around the cut score of 9.30% marks (equivalent to 2 SEMs) meaning that students falling below 47.70% would be reasonably certain to have failed. Roberts *et al*., suggest that even in a high stakes integrated assessment, consisting of written and objective structured clinical examinations (OSCE), well known issues of reliability suggest a more pragmatic 68% confidence (single SEM) around the pass/fail score should suffice where re-assessment is offered [37]. Given that the portfolio was not as high stakes as an end of medical program assessment, it was reasonable to ask students who had scored below 52.26% to undertake a supplementary assessment. Thus students’ scores were within a margin of error small enough so as to make the effort and use of resources of the assessment worthwhile [11]. In this case, further development was required to narrow the margin. In this iteration the assessment program was not reliable in the traditional sense. For a single portfolio and six assessment tasks, 11% of the variance in scores was due to true differences in the student. Twenty tasks would be needed to achieve summative reliability of 0.7, more than had been described in other settings [17].

The greatest source of measurement error in determining the absolute standard error of measurement related to context specificity, the observation that doing well on one task, would not necessarily mean doing well on another task. In portfolio assessment, this is to be expected given the diverse nature of the assessment tasks. However, given the homogeneity of the group, the latter finding may also relate to the degree with which students engaged with the varying tasks. Thus lower marks reflected the strategic effort they put into the tasks, rather than their true capability, if they had given equal priority to all the tasks. The next largest source of error related to rater subjectivity, an issue for rater training and rating task refinement.

We have considered the validity evidence as part of an integrative evaluative judgement [21, 36]. The portfolio assessment had content validity in that it was developed fit for purpose by local experts to sample the key features of the community placement learning outcomes, and several opportunities to promote student reflection. The pattern of inter task correlations is typical of similar research demonstrating the context specificity of students’ behaviours in the portfolio. For construct validity, the assessment was able to differentiate between contrasting levels of performance [23] using analysis of variance, over the four streams commensurate with their growing experience of medicine.

### Implications

As with other assessment tools, attention to all of the processes in the assessment could refine the assessment and feasibly reduce measurement error [38]. Understanding and accounting for the major contribution of context specificity is the priority to address, and is the reason why for example objective structured clinical examinations need to be long to be reliable. Successive assessment tasks in writing; the EBM task and the Community Profile Project may call for the same ability complex (holding down the student task interaction), whereas tasks in the clinical setting were assessing diverse constructs and therefore elevating the student task interaction [11].

From a learning and teaching perspective it is problematic calling context specificity a “measurement error” even though this may be correct from a psychometric point of view. For example, it is undesirable to match student learning tasks by ensuring they are testing similar things, thereby reducing context specificity, just to fit psychometric criteria. The strength of a clinical placement is that the student is immersed in their future work place, and will need to demonstrate many different skills. It is equally problematic trying to equalise tasks in terms of cognitive load [15], and engagement to students.

It was not considered feasible to increase the overall assessment burden on students by increasing the number of tasks, nor to convert currently unmarked tasks, for example, the formative self-reflective critical incident analysis into a summative element. Feedback from the academic assessors suggested that the academic assignments would benefit from reducing the overall number of task checklist items and more carefully discriminating between them through a clarification of the items. This action might impact on context specificity [19].

The implication of this discussion on context specificity is that for programmatic portfolios to be robust, the rules of measurement need to change so that teaching and learning is not subverted by psychometric rules. We need to be guided by an expert discussion based on the work of Cronbach *et al*. [11] as to how this might happen.

There were also student perceptions about the fairness of the weighting system, which prioritised academic assignments rather than the performance-based clinical supervisor ratings. Faculty had implemented the weighting, as they perceived that supervisor ratings were likely to be unreliable, given the number of GP supervisors (on average 80) that were involved in each rotation. Multivariate G-theory has been used elsewhere to estimate, for example, the composite reliability of an undergraduate clinical examination composed of several components, and the effect of item weighting and test length [39]. In this portfolio, there was little to be gained in the weighting system, as the addition of the raw percentage scores provided the lowest estimates of measurement error.

Addressing training assessors and tightening definitions in the marking criteria has traditionally been a way to address assessors’ subjectivity. However, neither strategy (alone or in combination) has resolved the persistent challenges of rater variability. There is thus a need for novel evidence-based approaches. Assessors may have used different schemas in judging student performance, in a process that has similarities to clinical reasoning [16]. By investigating the perceptual and processing capacities of our raters, and the schema they operate by, and then aligning rating tasks with raters (or assessment programs), we may be able to demonstrate improved discrimination between dimensions and/or students in future iterations of the assessment. This may also give a better sense of what variability should be treated as statistical “error” as opposed to meaningful differences in the ratings of experts [14–16].

The MCQ was the standout in terms of non-equivalence with the other portfolio tasks, as demonstrated by the different mean score and the standard deviation, and that it was machine marked. However it was considered by the Community placement committee to be an important driver of learning around common or important primary care presentations.

Cronbach *et al*. [11] provides a formula for calculating a whole of portfolio SEM derived from weightings of the individual tasks and the task SEM. We were unable to calculate the variance components of individual tasks, as we did not have access to the checklist items scores making up each task.

The portfolio-based learning included explicit and mandatory critical self-reflection [4, 5] and there was oral feedback from academics on student self-reflection in small group work [6, 9]. The requirement was one of participation, it seemed unhelpful to try and score this aspect.

To support student learning, assessors were also encouraged to give qualitative written feedback on the achievement of learning outcomes to individual students in the on-line marking system [10].

It is reasonable to assume that longer clinical placements; the so called longitudinal clinical clerkships, would offer greater justification for the use of portfolio, where a student might be placed for period of three months or more [26]. In the light of an international move towards assessment for learning [8, 40, 41], it is increasingly important to understand how assessment influences learning and teaching in clinical placements. Given the effort, resource issues, and challenging psychometric problems with programmatic portfolios it is important to demonstrate enhanced learning for students in clinical education, and this will require further research.

### Limitations of the study

As far as we are aware this is the first study to examine the generalisability of a portfolio designed as a programmatic assessment. This was a secondary analysis of assessment data derived from a programmatic portfolio. A curriculum committee designed the portfolio learning activities with a focus on enhancing student learning, yet maintaining the psychometric rigor demanded by the assessment strategy of the school. As is often the case in such settings there was no fully formalized design that assigned specific assessors or a specific number of assessors to each portfolio task. No assessor marked all portfolio tasks, but many assessors marked a number of tasks across students [35]. An alternative design, which was considered, was assessor nested within tasks crossed with students. This gave similar values for student, task and the student by task interaction. However, we chose the partially crossed model to best reflect this particular setting. Portfolios require a high level of resources in terms of learning support and for assessment [7, 9, 11]. In this iteration we were only able to collect the portfolio task total scores assigned to individual raters and not the individual checklist items that made up those scores. We were thus unable to determine within-task variance, and fully acknowledge the limitations of this data set in demonstrating the proper consideration of within and between task variance. We accept that the G study design, was simplistic, and may have over or underestimated some sources of error.

## Conclusion

A portfolio validated as a programmatic assessment of an integrated clinical placement has demonstrated reasonable measurement characteristics. We have demonstrated modest precision in assessing students’ achievement of a standard in primary and community care learning outcomes. Reliability is an unhelpful statistic in determining whether students have reached a certain standard and an absolute standard error of measurement is more appropriate. There were identifiable areas where measurement error could be reduced to provide more certainty around decision-making. Reducing the noise in the measurement would require engaging with the student body on the value of the tasks, more focussed academic and clinical supervisor training, and revisiting the rating tasks and the rubric of the assessment in the light of rater feedback. There are three rich areas for further research. First in encouraging an international consensus in how to demonstrate technical adequacy in reporting programmatic assessments such as a portfolio. Second, unpacking the extent to which the student task interaction and rater subjectivity can be shaped around newer understandings of context/case specificity and rater cognition. Third, understanding how aggregates of complex and diverse assessments enhance student learning. Programmatic portfolios will be particularly relevant to the international conversation about developing assessment of longitudinal integrated clinical placements (LICs).
